# Proteolysis-inducing factor is expressed in tumours of patients with gastrointestinal cancers and correlates with weight loss

**DOI:** 10.1054/bjoc.2001.1830

**Published:** 2001-06

**Authors:** R Cabal-Manzano, P Bhargava, A Torres-Duarte, J Marshall, P Bhargava, I W Wainer

**Affiliations:** Lombardi Cancer Center, Georgetown University Medical Center, 3800 Reservoir Road NW, Washington DC 20007; Division of Clinical Pharmacology, Georgetown University Medical Center, 3900 Reservoir Road NW, Washington DC 20007; Department of Pathology, Georgetown University Medical Center, 3900 Reservoir Road NW, Washington DC 20007; Department of Pharmacology, Georgetown University, 3900 Reservoir Road NW, Washington DC 20007

**Keywords:** proteolysis-inducing factor, tumour expression, weight-loss, humans

## Abstract

Proteolysis-inducing factor (PIF), a novel cachectic factor, is detectable in the urine of cancer patients experiencing weight loss. We report the expression of PIF in gastrointestinal cancers, and a correlation between PIF expression in tumours, its detection in urine and weight-loss. These data provide the first direct evidence that tumours are the source of PIF in humans. © 2001 Cancer Research Campaign http://www.bjcancer.com


British Journal of Cancer (2001) 84(12), 1599–1601
© 2001 Cancer Research Campaign
doi: 10.1054/ bjoc.2001.1830, available online at http://www.idealibrary.com on IDEAL®
http://www.bjcancer.com

Short Communication

### Proteolysis-inducing factor is expressed in tumours of patients with gastrointestinal cancers and correlates with weight loss

**R Cabal-Manzano[1], P Bhargava[1,2], A Torres-Duarte[4], J Marshall[1], P Bhargava[3] and IW Wainer[4]**

[1]Lombardi Cancer Center, Georgetown University Medical Center, 3800 Reservoir Road NW, Washington DC 20007; [2]Division of Clinical Pharmacology, Georgetown University Medical Center, 3900 Reservoir Road NW, Washington DC 20007; [3]Department of Pathology, Georgetown University Medical Center, 3900 Reservoir Road NW, Washington DC 20007; [4]Department of Pharmacology, Georgetown University, 3900 Reservoir Road NW, Washington DC 20007

**Summary** Proteolysis-inducing factor (PIF), a novel cachectic factor, is detectable in the urine of cancer patients experiencing weight loss. We report the expression of PIF in gastrointestinal cancers, and a correlation between PIF expression in tumours, its detection in urine and weight-loss. These data provide the first direct evidence that tumours are the source of PIF in humans. © 2001 Cancer Research Campaign http://www.bjcancer.com

**Keywords**: proteolysis-inducing factor; tumour expression; weight-loss; humans

Cachexia, a syndrome characterized by weight loss, anorexia, weakness and asthenia, may be seen in up to 80% of patients with gastrointestinal cancers (Bruera, 1997). Cachexia and weight loss are associated with impaired quality of life and reduced survival in patients with advanced cancer (Dewys et al, 1980).

Proteolysis-inducing factor (PIF) was identified as a tumour product in the circulation of mice bearing a cachexia-inducing tumour but not in mice bearing a tumour that does not induce cachexia (Todorov et al, 1996b). PIF, a glycoprotein of relative molecular mass 24 kDa, caused weight loss by inducing enhanced protein degradation without decreasing the appetite in mice. PIF was also found to be present in the urine of cachectic cancer patients while being absent from normal subjects, individuals with weight loss due to trauma and cancer patients with little or no weight loss (Todorov et al, 1996a). However, human tumours have not been shown to express PIF, and tumour expression of PIF has not been correlated with weight loss in humans. This study was conducted to examine these important issues linking PIF to cachexia in humans.

### MATERIALS AND METHODS

### Patients

We studied 16 tumour samples from patients with gastrointestinal cancers who had undergone surgery or biopsy at the Lombardi Cancer Center, Georgetown University Medical Center. The samples consisted of formalin-fixed, paraffin-embedded tissue, of which 4–5 µm sections were obtained for analysis. For most patients, only tumours from metastatic sites were available. Medical records were reviewed to determine the type and stage of cancer at the time of diagnosis, and to obtain serial weight measurements.

*Received 9 November 2000*
*Revised 22 February 2001*
*Accepted 28 February 2001*

### Urine samples

5 ml aliquots of urine were collected from all patients on the study. The samples were concentrated (50x), dialysed (cut off 18 kDa) at 4˚C using Minicon B15 concentrator (Millipore Corp, Burlington, MA) to remove low-molecular mass species, and further diluted in water for analysis.

### Immunohistochemistry

Tissue sections were deparaffinized, hydrated, and heated in a citrate buffer (10 mM, pH 6.0) at 90˚C for 30 min. Nonspecific reactivity was blocked by incubation with 10% normal horse serum for 30 minutes, followed by addition of the anti-PIF antibodies (generously provided by Dr Michael Tisdale, Aston University, Birmingham, UK) at a concentration of 25 µg $ml^{-1}$. The tissue sections were incubated overnight at 4˚C. After multiple PBS washes, tissue sections were incubated for 20 min at room temperature with biotinylated anti-mouse IgG antibody made in goat. The detection system was Avidin–Biotin Complex (ABC, Vector Labs Inc, Burlingame, CA) which was incubated with the tissue for 30 min at 37˚C. After multiple washes, peroxide activity was visualized after reaction with diaminobenzamine followed by nuclear counter staining with haematoxylin, dehydration in ETOH gradients, xylene and finally mounting under coverslips. The negative controls were tissue without primary antibody, an irrelevant negative antibody and a mouse IgG2a, kappa (UPC 10) purified immunoglobulin at the same concentration as anti-PIF antibody. All tissue sections were evaluated for PIF expression by 2 investigators. Tumour, epithelial and mesenchymal cells, and the extracellular matrix were evaluated for PIF staining. The percentage of stained cell were taken as a measure of PIF expression. Tissue was considered positive for PIF expression if a granular staining pattern was found in the cytoplasm of more than 30% cells at a magnification of 100×

RCM and PB contributed equally to this work.

### Urine assay for PIF

Urine samples were analysed by capillary electrophoresis, and structural assignments were made by Western blot analyses using antibodies raised against murine PIF and cross validated with off-line matrix assisted laser desorption ionization time of flight (MALDI TOF) mass spectrometry as previously described (Choudhary et al, 1999). Briefly, the separation was performed on a 64.5 cm × 75 µm ID fused-silica capillary column at an applied voltage of 15 kV with 60 mM sodium tetraborate, pH 9.2 as the buffering electrolyte. The peak eluting at 15 min could be identified as PIF. Micropreparative CE was performed and the fraction containing the factor was collected for off-line analysis by MALDI-TOF. Typically, 50–100 laser shots were summed for each spectrum. Mass calibration was performed using a protein standard (G-2053A, Hewlett Packard) comprising myoglobin, cytochrome C and bovine serum albumin.

### RESULTS

PIF staining was detectable in the tissue from 6 patients (2 colon, 1 pancreatic, 2 gastric, 1 cholangio-carcinoma) and appeared as granular cytoplasmic staining within cells. The staining was observed in the cytoplasm of tumour cells in 5 patients, and in the cytoplasm of epithelial and mesenchymal cells surrounding the tumour in 1 patient. The number of stained cells in tumours varied from 30% to 80% positive cells. Staining was seen in clusters of tumour cells as well as single malignant cells scattered in the parenchyma. Moderate to poorly differentiated carcinomas showed stronger staining compared with well-differentiated carcinomas. Representative positive and negative results for tumour cells are shown in Figure 1(A). The single case of non-tumoral staining was observed in approximately 60% of mucosal and periglandular cells in a patient with gastric carcinoma (Figure 1C). Tumour cells in this case showed faint staining for PIF in < 20% of cells (data not shown). The 2 investigators had < 8% discrepancy between estimates of the number of stained cells in tissue sections.

Urine for PIF was collected at varying time intervals following diagnosis. All patients with positive staining for PIF in their tissue samples had detectable PIF in the urine as shown in Table 1. PIF was detectable as a ~24 kD protein eluting at 15 minutes, and was identified using Western blot analysis employing antibodies raised against PIF isolated from a murine adenocarcinoma, MAC-16. PIF was detectable in urine up to 2 years following diagnosis in patients who had positive staining in the tissue biopsy. A variety of gastrointestinal carcinomas were studied, and the site of the primary tumour had no effect on the presence of PIF. The detection of PIF in tissue and urine was also not dependent upon the presence of secondary metastatic tumours.

There was strong correlation between the presence of PIF in tumour and urine, and weight loss. All 6 patients whose tissues and urine tested positive for PIF had experienced weight loss between 8% and 39% of the pre-illness weight (average 18%),

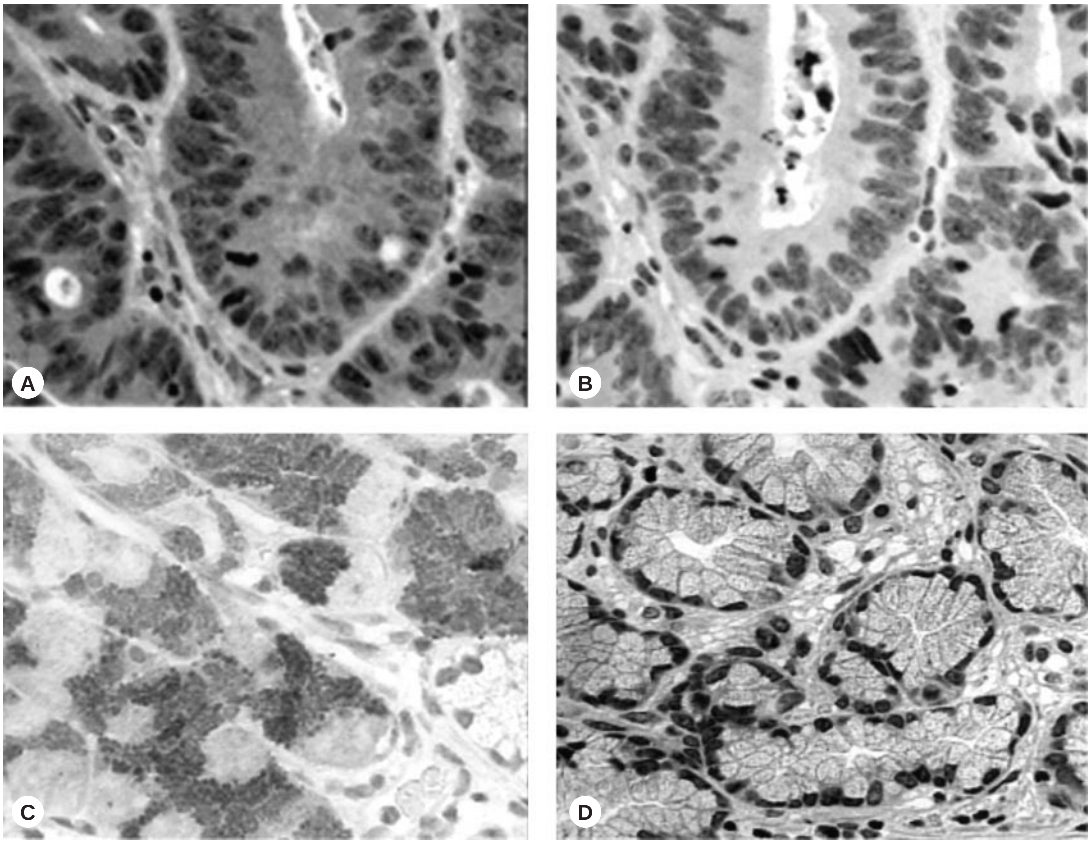

**Figure 1** (**A**) Colorectal carcinoma showing expression of PIF in the cytoplasm of tumour (immunohistochemistry, x400). (**B**) Negative control. (**C**) Gastric glands showing expression of PIF in glandular epithelial and periglandular cells (Immunohistochemistry, x400). (**D**) Negative control.

*© 2001 Cancer Research Campaign*

**Table 1** Comparison PIF expression in tumour tissue and urine, and comparison with weight loss in patients with gastrointestinal carcinomas

| Patient No. | Primary tumour | Tissue PIF staining | | | Urine PIF | | Weight loss (%) |
|---|---|---|---|---|---|---|---|
| | | Date | Biopsy site | Result | Date of collection | Result | |
| 1 | Colon | 4/98 | Colon | (–) | 4/98 | (–) | 7 |
| 2 | Colon | 2/97 | Liver | (–) | 2/99 | (–) | 4 |
| 3 | Colon | 7/97 | Colon | (+) | 2/98 | (+) | 9 |
| 4 | Colon | 7/96 | Lung | (+) | 12/97 | (+) | 24 |
| 5 | Pancreas | 7/98 | Lymph Node | (–) | 7/98 | (–) | 4 |
| 6 | Pancreas | 3/97 | Liver | (–) | 3/98 | (–) | 15* |
| 7 | Pancreas | 2/98 | Ascitic Fluid Cells | (–) | 9/98 | (–) | 6 |
| 8 | Pancreas | 6/98 | Pancreas | (+) | 6/98 | (+) | 8 |
| 9 | Gastric | 7/97 | Peritoneal Deposit | (–) | 7/97 | (–) | 3 |
| 10 | Gastric | 10/96 | Esophagus | (–) | 3/98 | (–) | 5 |
| 11 | Gastric | 3/98 | Esophagus | (–) | 3/98 | (–) | 6 |
| 12 | Gastric | 9/98 | Esophagus | (–) | 9/98 | (–) | 1 |
| 13 | Gastric | 11/98 | Liver | (+) | 11/98 | (+) | 14 |
| 14 | Gastric | 4/98 | Esophagus | (+) | 4/98 | (+) | 39 |
| 15 | Small Bowel | 1/98 | Abdominal Wall | (–) | 1/98 | (–) | 22** |
| 16 | Cholangio-carcinoma | 12/98 | Liver | (+) | 12/98 | (+) | 18 |

* Biliary obstruction, ** intestinal obstruction.

while 8 patients whose tissues and urine tested negative for PIF had experienced weight loss of between 1% and 7% (average 5%). 2 remaining patients who had also tested negative for PIF in tissue and urine samples, had experienced weight loss that was attributed to concomitant medical problems that impaired food intake (weight loss of 15% and 22% of the pre-illness weight, attributed to biliary obstruction and intestinal obstruction, respectively).

### DISCUSSION

The identification of proteolysis-inducing factor (PIF) in the urine of cachectic patients, and correlation of its presence with weight loss have helped establish PIF as a novel cachectic factor in humans (Todorov et al, 1996a; Wigmore et al, 2000). However, these studies have been criticised because the authors identified PIF only in the urine and not in the serum (Gullu and Marangoz, 1999). Moreover, the source of PIF in cachectic patients was unknown. It was thought that human tumours, like mouse tumours, produce PIF but direct evidence of PIF expression in tumours was lacking. Our data show that PIF is expressed in tumours and mesenchymal tissue of patients who have PIF detectable in the urine, and is strongly associated with weight loss in cancer patients. The low abundance of PIF in serum (about 40 ppb) would require extensive prepurification for detection of the factor, and has been the primary obstacle in using serum measurements for studies (Tisdale, 1999). While we did not measure PIF in serum, the detection of PIF in tumours and urine of the same patients implies its transport through the bloodstream.

While PIF was expressed predominantly in tumour tissue, it was interesting to note that one tumour biopsy showed PIF expression in mucosal and periglandular cells situated close to the tumour. This could suggest several possibilities: PIF may be an inducible factor, and may be expressed in surrounding epithelial and mesenchymal cells under the influence of tumour cells. Alternatively, PIF could be a secreted factor that is stored in the cells surrounding the tumour. Further studies will be needed to confirm this observation and to identify the mechanisms of production and secretion of PIF.

In conclusion, data from our study show that tumours are the source of PIF in patients with gastrointestinal cancers, and the expression of PIF in tumours correlates directly with its presence in urine. These data are also consistent with prior studies correlating the presence of PIF in the urine with weight loss in cancer patients. We are prospectively evaluating tumour, serum and urine PIF as a markers of cachexia and disease prognosis in patients with gastrointestinal cancers.

### REFERENCES

Bruera E (1997) Anorexia, cachexia, and nutrition. *BMJ* **315**: 1219–1222

Choudhary G, Chakel J, Hancock W, Torres-Duarte A, McMahon G and Wainer I. W (1999) Investigation of the potential of capillary electrophoresis (CE) with off-line matrix assisted laser desorption/ionization time of flight (MALDITOF) mass spectrometry for clinical analysis: examination of a glycoprotein factor associated with cancer cachexia. *Anal Chem* **71**: 855–859

Dewys WD, Begg C, Lavin PT, Band PR, Bennett JM, Bertino JR, Cohen MH, Douglass HO, Jr, Engstrom PF, Ezdinli EZ, Horton J, Johnson GJ, Moertel CG, Oken MM, Perlia C, Rosenbaum C, Silverstein MN, Skeel RT, Sponzo RW and Tormey DC (1980) Prognostic effect of weight loss prior to chemotherapy in cancer patients. Eastern Cooperative Oncology Group. *Am J Med* **69**: 491–497

Gullu I and Marangoz S (1999). Induction of cachexia in mice (letter). *Br J cancer* **79** : 1620.

Tisdale M (1999). Induction of cachexia in mice-reply (letter). *Br J Cancer* **79** : 1620.

Todorov P, Cariuk P, McDevitt T, Coles B, Fearon K and Tisdale M (1996a) Characterization of a cancer cachetic factor. *Nature* **379** : 739–742

Todorov PT, McDevitt TM, Cariuk P, Coles B, Deacon M and Tisdale MJ (1996b) Induction of muscle protein degradation and weight loss by a tumour product. *Cancer Res* **56**: 1256–1261

Wigmore SJ, Todorov PT, Barber MD, Ross JA, Tisdale MJ and Fearon KC (2000) Characteristics of patients with pancreatic cancer expressing a novel cancer cachectic factor. *Br J Surg* **87**: 53–58.

*© 2001 Cancer Research Campaign* 

